# All-Organic Quantum Dots-Boosted Energy Storage Density in PVDF-Based Nanocomposites via Dielectric Enhancement and Loss Reduction

**DOI:** 10.3390/polym17030390

**Published:** 2025-01-31

**Authors:** Ru Guo, Xi Yuan, Xuefan Zhou, Haiyan Chen, Haoran Xie, Quan Hu, Hang Luo, Dou Zhang

**Affiliations:** 1 Powder Metallurgy Research Institute, State Key Laboratory of Powder Metallurgy, Central South University, Changsha 410083, China; ruguo@cuhk.edu.hk (R.G.); haoranxie123@163.com (H.X.); quanhuqq@163.com (Q.H.); douzhang@csu.edu.cn (D.Z.); 2 Department of Mechanical and Automation Engineering, The Chinese University of Hong Kong, Shatin, Hong Kong, China; 3 College of Chemistry and Chemical Engineering, Central South University, Changsha 410083, China; xiyuan@csu.edu.cn; 4 College of Energy and Power Engineering, Changsha University of Science and Technology, Changsha 410114, China

**Keywords:** dielectric capacitor, PVDF polymer, energy density, carbon quantum dots, crystallization behavior

## Abstract

Dielectric capacitors offer immense application potential in advanced electrical and electronic systems with their unique ultrahigh power density. Polymer-based dielectric composites with high energy density are urgently needed to meet the ever-growing demand for the integration and miniaturization of electronic devices. However, the universal contradictory relationship between permittivity and breakdown strength in traditional ceramic/polymer nanocomposite still poses a huge challenge for a breakthrough in energy density. In this work, all-organic carbon quantum dot CDs were synthesized and introduced into a poly(vinylidene fluoride) PVDF polymer matrix to achieve significantly boosted energy storage performance. The ultrasmall and surface functionalized CDs facilitate the polar *β*-phase transition and crystallinity of PVDF polymer and modulate the energy level and traps of the nanocomposite. Surprisingly, a synergistic dielectric enhancement and loss reduction were achieved in CD/PVDF nanocomposite. For one thing, the improvement in *ε*_r_ and high-field *D*_m_ originates from the CD-induced polar transition and interface polarization. For another thing, the suppressed dielectric loss and high-field *D*_r_ are attributed to the conductive loss depression via the introduction of deep trap levels to capture charges. More importantly, *E*_b_ was largely strengthened from 521.9 kV mm^−1^ to 627.2 kV mm^−1^ by utilizing the coulomb-blockade effect of CDs to construct energy barriers and impede carrier migration. As a result, compared to the 9.9 J cm^−3^ for pristine PVDF, the highest discharge energy density of 18.3 J cm^−3^ was obtained in a 0.5 wt% CD/PVDF nanocomposite, which is competitive with most analogous PVDF-based nanocomposites. This study demonstrates a new paradigm of organic quantum dot-enhanced ferroelectric polymer-based dielectric energy storage performance and will promote its application for electrostatic film capacitors.

## 1. Introduction

The rapid development of renewable and sustainable energy technology poses an urgent requirement for efficient, low-cost, and environmentally friendly electric energy storage systems. Compared with electrochemical energy storage devices, electrostatic capacitors that possess unique merits of ultrahigh power density (~10^7^ W kg^−1^), an ultrafast energy release ability (<0.01 s), and high voltage tolerance (>1000 V), have been widely used in advanced power electronics and pulsed power systems [1,2,3]. Specially, polymer-based film capacitors with the intrinsic advantages of lightweight, facile processability, low dielectric loss, and high breakdown strength showcase enormous application prospects [4,5]. However, the low energy storage density restricts their widespread application [6]. For instance, the state-of-the-art commercially available film capacitor with biaxially oriented polypropylene (BOPP) shows a limited energy density of 2~4 J/cm^3^ [7]. Accordingly, a large assembly volume and huge weight for the system are required to meet energy requirements in practical applications, which is contrary to the ever-growing demands of integration and miniaturization for modern electronic devices [8]. Developing polymer-based dielectrics with high energy density has become a research hotspot and critical challenge in recent decades.

The effective energy density of dielectrics is determined through the following formula:

(1) $$U_{dis}=\int_{D_{r}}^{D_{m}}EdD$$

where *E* is the dielectric strength, and *D*_m_ and *D*_r_ are the maximum and remnant displacement. For linear dielectrics, this equation can be expressed as follows:

(2) $$U_{dis}=1/2\varepsilon_{0}\varepsilon_{r}E^{2}$$

where *ε*_0_ and *ε*_r_ mean the vacuum and relative dielectric constant. Therefore, both *ε*_r_ and *E*_b_ are critical factors for the improvement of energy density [9]. Among various polymer dielectrics, poly(vinylidene fluoride) (PVDF), possessing a high dielectric constant (~8 at 1 kHz) originating from the strong C-F dipoles and spontaneous orientation, has become a preferred polymer, and it attracts strong research interest [10,11,12,13]. Most recently, the composite strategy of incorporating ceramic nanofillers (e.g., BaTiO_3_, TiO_2_, etc.) into a PVDF polymer matrix has proven to improve the dielectric property and capacitive performance [14,15,16]. However, a significant enhancement in *ε*_r_ (e.g., 10 times that of the polymer matrix) often relies on the high loading ratio of nanofillers (>10 vol.%), which inevitably sacrifices the breakdown strength, *E*_b_, resulting in a considerable energy loss [17,18,19]. For one thing, the large dielectric mismatch between nanofillers and a polymer matrix could cause concentrated electric field distribution to trigger a breakdown discharge [20,21,22,23]. For the other thing, overloading-induced nanofiller agglomeration also causes uneven and incompatible interfacial regions, leading to a sharp increase in the leakage current and a reduction in *E*_b_ [24,25]. Therefore, exploring novel nanofillers to settle the above contradictory relationship between *ε*_r_ and *E*_b_ is still highly desired.

Quantum dots, as a newly rising zero-dimensional material, have become a kind of ideal candidate for optoelectronic devices and energy storage applications [18,26,27]. With the unique characteristics of an ultrasmall size (less than 10 nm), a large specific surface area, and easy surface functionalization, they hold great potential to serve as functional fillers in boosting the energy storage performance of polymer composites [28,29]. Recently, a sandwiched polymer/metal architecture with Au nanodots was constructed based on polycarbonate (PC) matrix films, exhibiting a high energy density of 6.25 J cm^−3^ at 150 °C [30]. The effectively suppressed breakdown and leakage current of this composite under high fields benefited from the deep traps and “Coulomb islands” at interlayered interfaces introduced via discontinuous Au dots. Additionally, perovskite Cs_2_SnI_6_ quantum dots were introduced into a polyetherimide PEI polymer with robust electrostatic attraction to trap the injected and excited electrons; the conduction loss was largely decreased, yielding an energy density of 7.2 J/cm^3^ at 350 MV/m and 100 °C in the composite film [28]. Wang et al. incorporated cadmium-based quantum dots (CdSe/Cd_1−x_Zn_x_S) into poly(methyl methacrylate) PMMA polymer [18]. The significant dielectric enhancement was achieved because of polarization’s contribution from quantum dots, which induced a larger interphase region, yielding a high energy density of 17.6 J cm^−3^ in this nanocomposite.

Compared with the above inorganic quantum dots, all-organic carbon quantum dots (CDs) with abundant surface functional groups are expected to form tighter and more compatible interface regions to improve interface polarization while maintaining a high breakdown strength. Most recently, CD-enhanced dielectric composites have mainly been based on linear polymer matrices for high-temperature energy storage, such as polyetherimide or PEI [31,32], fluorene polyester or FPE [33], and polyphenylene sulfone (PPSU) [34]. In comparison, as a typical ferroelectric polymer, PVDF, possesses a semi-crystalline nature and exhibits a complex structure with multiple crystalline polymorphs (*α*, *β*, *γ*, *δ*, and *ε* phases) and amorphous regions [35]. However, research on the effect of CDs on a ferroelectric polymer-based dielectric nanocomposite has not been carried out. In particular, CD-induced crystallization behavior and its effect on electrical properties is still ambiguous and urgently needs to be clarified.

In this work, all-organic carbon quantum dots were synthesized and introduced into a PVDF polymer matrix to achieve the significantly boosted energy storage performance of dielectric nanocomposites. The ultrasmall and surface functionalized CDs facilitate the polar *β*-phase transition and crystallinity of the PVDF polymer. Additionally, the appropriate introduction of CDs effectively modulates the energy level and traps of the nanocomposite, leading to an increased energy band gap, *E*_g_. Surprisingly, a synergistic dielectric enhancement and loss reduction were achieved in the CD/PVDF nanocomposite. For one thing, the improvement in *ε*_r_ and high-field *D*_m_ originated from the CD-induced polar transition and interface polarization. For another thing, the suppressed dielectric loss and high-field *D*_r_ were attributed to the conductive loss depression through the introduction of deep trap levels to capture charges. More importantly, *E*_b_ was largely increased from 521.9 kV mm^−1^ to 627.2 kV mm^−1^ by utilizing the coulomb-blockade effect of CDs to construct a high energy barrier and impede carrier migration. As a result, compared to the 9.9 J cm^−3^ for pristine PVDF, the highest discharge energy density of 18.3 J cm^−3^ was yielded in the 0.5 wt% CD/PVDF nanocomposite, which is competitive with most analogous PVDF-based nanocomposites. This study demonstrates a new paradigm of organic quantum dot-enhanced ferroelectric polymer-based dielectric energy storage performance, and it will promote this material’s application for electrostatic film capacitors.

## 2. Experimental Section

### 2.1. Raw Materials

All NaOH, acetaldehyde (40%), HCl (37%), N, and N-dimethylformamide (DMF, 99.5%) were purchased from Sinopharm Chemical Reagent Beijing Co., Ltd. (Beijing, China). The polyvinylidene difluoride (PVDF) powder was purchased from Polyk Technologies, Beijing, China.

### 2.2. The Synthesis of CD Powder

The CDs were synthesized following a reported simple chemical method. Firstly, a certain amount of NaOH was dissolved in deionized water, and then the solution was added to 200 mL of acetaldehyde aqueous solution, followed by vigorous stirring for 2 h. Secondly, the above solution was separated via centrifugation and washed with 1M of HCl solution and deionized water successively. The produced CD powders were finally obtained after drying in a vacuum oven for 12 h.

### 2.3. The Fabrication of CD/PVDF Nanocomposite Films

A certain amount of CD powder was weighted and added into the DMF solvent, followed by a 20 min ultrasonic treatment to evenly disperse the CD fillers. The PVDF powder was dissolved in the above solution followed via stirring at 50 °C for 12 h to obtain a homogeneous, light yellow solution. The solution was placed in a vacuum chamber and repeatedly vacuumed several times to remove tiny bubbles from the solution. Then, the above solution was poured onto a clear glass substrate, followed by tape casting using a scraper with a clearance of 400 μm. After drying at 80 °C for 48 h to evaporate solvent, CD/PVDF nanocomposites films with an approximate thickness of 15 µm were obtained.

### 2.4. Characterization and Instrumentation

X-ray diffraction (XRD, D/max 2550, Tokyo, Japan), a Fourier-transform infrared spectrophotometer (FTIR, Is-50, Thermo Fisher, Waltham, MA, USA), and X-ray photoelectron spectroscopy (XPS, AXIS SUPRA+, Belfast, UK) were used to characterize the crystalline phase of the nanocomposite. The average crystallite size was evaluated according to the Scherrer equation:

(3) $$D_{hkl}=K\lambda /\beta_{hkl}cos\theta$$

where *D*_hkl_ is the mean crystallite size along the [*hkl*] crystal plane, *β*_hkl_ is the full width at the half-maximum intensity (FWHM) measured on the diffraction profile, *K* is the shape factor with a value of 0.89, and *λ* is the wavelength of the incident X-rays [36]. The thickness of the CD/PVDF nanocomposite was characterized using a scanning electron microscope (SEM, Nova NanoSEM230, Bethel, CT, USA). The DSC experiments were conducted using a Thermal Analysis (TA) Instruments Q2000 calorimeter (Delaware, USA) under a nitrogen atmosphere with two heating runs. The first circle of heating (10 °C min^−1^) and cooling (10 °C min^−1^) was conducted to remove residual solvents and erase the thermal history of the polymer during the fabrication process. Then, DSC spectra during the second heating (10 °C min^−1^) were measured and used to analyze the crystallization behavior of the sample films. The calculation of crystallinity (*χ*_c_) followed the formula below:

(4) $$\chi_{c}={\Delta H}_{m}/{\Delta H}_{m}^{0}\times 100\%$$

where Δ*H_m_* and Δ*H_m_*^0^ are the melting enthalpies of the nanocomposite and PVDF polymer with 100% crystallinity (104.5 J g^−1^ for the PVDF), respectively. FTIR was used to investigate the crystalline phase transformation of the nanocomposites. The *β*-phase content was calculated according to the following equation:

(5) $$F\left(\beta \right)=A_{\beta }/\left({(K}_{\beta }/K_{\alpha })A_{\alpha }+A_{\beta }\right)\times 100\%$$

where *A_α_* and *A_β_* are the absorbance values at 764 and 840 cm^−1^, respectively. *K_α_* and *K_β_* are the corresponding absorbance coefficients, i.e., 6.1 × 10^4^ and 7.7 × 10^4^ cm^2^ mol^−1^, respectively [25]. The energy bandgap, *E_g_*, of the nanocomposite was analyzed using the Tauc plot method from a UV-vis absorption test (Shimadzu UV-3600i Plas, San Jose, CA, USA). The *E_g_* was determined according to the following equation:

(6) $${\left(\alpha h\nu \right)}^{2}=A(h\nu -E_{g})$$

where α is the absorbance index, h is Planck’s constant, v is the frequency, and A is a constant of 2 for a semiconductor [31].

### 2.5. Electrical Measurement

Au electrodes were sputtered on both sides of all the prepared sample films with magnetron sputtering using a mask with 2 mm-diameter eyelets. The dielectric properties of the films were tested using an LCR meter with a frequency range from 1 kHz to 10 MHz (Agilent 4294, Santa Clara, CA, USA). The electric displacement–electric field hysteresis loop (*D-E* loop) and leakage–electric field (*I–V*) curves were measured using a TF analyzer 2000 ferroelectric polarization tester (aixACCT, Aachen, Germany) at 10 Hz and room temperature. The dielectric breakdown field was tested by gradually increasing the applied field of 20 kV mm^−1^ each time until a breakdown occurred. The energy storage density and efficiency were calculated based on the *D–E* loops.

## 3. Results and Discussion

### 3.1. Microstructural Characterization of CD Powders

**Figure 1:**
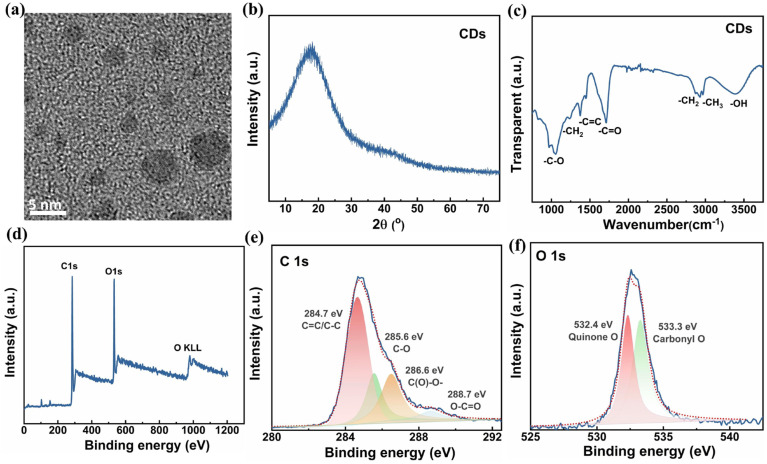
Microstructural characterization of CD powders. (**a**) High-resolution TEM image, (**b**) XRD pattern, (**c**) FTIR spectrum, (**d**) XPS full survey spectrum, (**e**) C1s, and (**f**) O1s spectra of CD powders.

The high-resolution TEM image in Figure 1a shows the monodisperse spherical particles of CDs with a diameter of about 3~5 nm. Additionally, a broad peak at ~17.0° was exhibited in the XRD pattern, indicating a disordered carbonaceous structure of CDs (Figure 1b). The molecular structure characterized by the FTIR spectrum demonstrates the multiple functional groups, i.e., a carbon–carbon double bond (C=C, 1374 cm^−1^), hydroxyl (-OH, 3380 cm^−1^), and a carbonyl double bond (C=O, 1715 cm^−1^), etc., adsorbed on CDs’ surface during their chemical preparation (Figure 1c). Furthermore, in Figure 1d, the XPS full survey confirms the presence of C and O elements. In particular, four characteristic peaks located at 284.7 eV, 285.6 eV, 286.6 eV, and 288.7 eV and corresponding to C=C/C-C, C-O, C(O)-O-, and O-C=O bonds, respectively, were observed in the high-resolution C1s spectrum (Figure 1e) [33]. Two characteristic peaks at 532.4 eV and 533.3 eV represent the quinone O and carbonyl O, respectively, in the O1s spectrum (Figure 1f) [37]. These results proved that the surface of CDs contains a carbon and rich oxygen-containing functional group, which will facilitate the formation of O-H-type hydrogen bonds and favor the interfacial compatibility of CDs with a PVDF polymer matrix.

### 3.2. Structural Characteristics of CD/PVDF Nanocomposite Films

**Figure 2:**
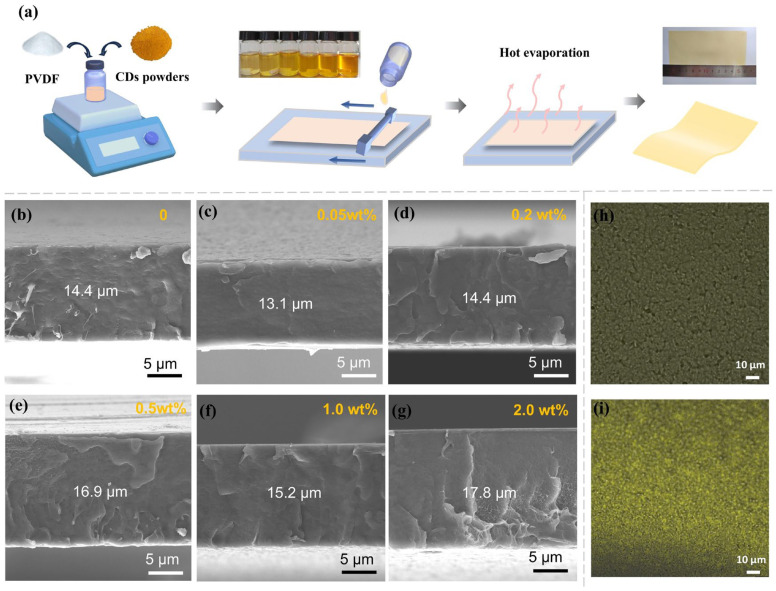
(**a**) Process diagram for preparing CD/PVDF nanocomposite film through the solution-casting method. Cross-sectional SEM image of nanocomposite with different CD content: (**b**) 0, (**c**) 0.05 wt%, (**d**) 0.2 wt%, (**e**) 0.5 wt%, (**f**) 1.0 wt%, and (**g**) 2.0 wt%. LSCM images of the (**h**) pristine PVDF film and (**i**) 2 wt% CD/PVDF nanocomposite.

Firstly, Figure 2a demonstrates the process diagram for preparing CD/PVDF nanocomposite film through a solution-casting method; the inset clearly shows the homogeneous CD/PVDF solutions with different CDs. Benefiting from its ultrafine size and surface functionalization, the CDs exhibit excellent dispersibility in the solution without any CD precipitation or aggregation to fabricate the high-quality CD/PVDF nanocomposite film. Additionally, the cross-sectional SEM images of CD/PVDF nanocomposite films with varied CD weight ratios from 0 to 2.0 wt% are shown in Figure 2b–g. The film thickness was uniformly controlled at the range of 13~18 μm. The CD content reached a high level of 2.0 wt%; there are no obvious nanofiller agglomeration defects inside the nanocomposite film, indicating that CDs have good interfacial compatibility with the PVDF matrix. Due to the quantum size of CDs, it is difficult to directly observe their distribution state in the PVDF matrix even under high-resolution SEM. Given that CDs possess unique photoluminescent features, the laser confocal microscope image (LSCM) was adopted to characterize the dispersibility of CDs in the nanocomposite. At 488 nm-wavelength excitations, the 2 wt% CD/PVDF nanocomposite demonstrated uniformly scattered yellow fluorescent dots, compared with no fluorescence for the pristine PVDF film (Figure 2h,i). Differing from the conventional ceramic–polymer composites with inevitably weak interfacial interactions, these organic CDs possess excellent dispersibility and interface compatibility within the polymer matrix, which can greatly preserve the advantages of the mechanical properties and processing performance of dielectric films.

**Figure 3:**
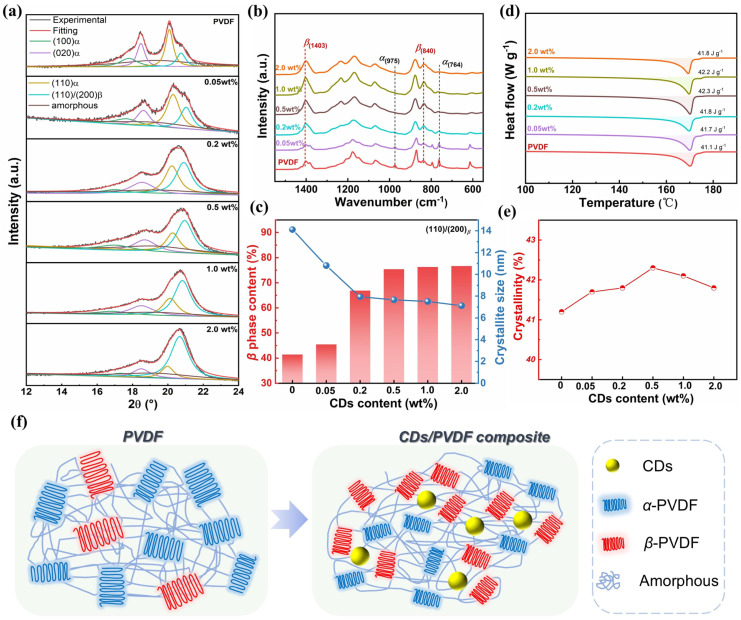
(**a**) XRD pattern, (**b**) FTIR spectra, (**c**) calculated phase content and crystallite size, (**d**) DSC thermographs, and (**e**) calculated crystallinity of the CD/PVDF nanocomposite with different CD contents. (**f**) Schematic diagram of CD-induced crystallization behavior evolution.

The crystallization behavior of the CD/PVDF nanocomposite with different CD content is further discussed here. As is well known, PVDF is a semi-crystalline polymer, and it has complex structures of multiple crystalline polymorphs (α, *β*, γ, δ, and ε phase), along with the existence of amorphous regions. Among these, the α phase adopts a trans-gauche-trans-gauche′ (TGTG′) conformation with lower polarity. In contrast, the polar *β* phase, characterized by an all-trans (TTTT) conformation, features uniformly aligned dipole moments and substantial ferroelectric domains. The peak fitting results of XRD in Figure 3a show that, with increasing CD content, the intensities of the diffraction peaks belonging to (100)*_a_*-PVDF, (020)*_a_*-PVDF, and (110)*_a_*-PVDF gradually weaken while accompanied by an obvious increase in the (110/200)*_β_*-PVDF peak intensity, indicating a polar crystalline phase transition in CD/PVDF nanocomposites. Additionally, the FTIR results in Figure 3b also confirmed this phenomenon, in which the characteristic peaks of the polar *β*-phase at 840 cm^−1^, 1279 cm^−1^, and 1403 cm^−1^ were enhanced, while those of the nonpolar α-phase at 762 cm^−1^ and 974 cm^−1^ were reduced simultaneously. Specifically, the quantified phase content and crystallite size are given in Figure 3c. Largely increased *β* phase content was observed from 41.4% for the pristine PVDF film to 76.6% for the 2 wt% CD/PVDF nanocomposite. The introduction of CDs also significantly decreased the crystalline size of the (110)/(200)*_β_*, e.g., from 14.1 nm for the pristine PVDF film to 7.12 nm for the 2 wt% CD/PVDF nanocomposite. The CD-induced polar phase transition could be attributed to the following reason: First, the ultrasmall size of CDs acting as the nucleating agent could induce local molecular chain movement, leading to its conformational rearrangement from trans-gauche-trans-gauche′ (TGTG′, *α* phase) to an all-trans planar zigzag (TTTT, *β* phase). Second, the surface functionalization of CDs enables a strong electrostatic interaction between abundant oxygenous functional groups and the fluorine atom on the PVDF polymer, thus driving the dipole arrangement to form a *β*-phase. In Figure 3d, DSC traces as a function of temperature are examined to assess the crystallinity of the CD/PVDF nanocomposite. As is shown in Figure 3e, an improvement in crystallinity from 41.2% for the pristine PVDF film to 42.3 for the 0.5 wt% CD/PVDF nanocomposite was obtained, indicating that the introduction of CDs promotes some amorphous molecules’ transformation into crystalline structures. Finally, a schematic diagram of the microstructure evolution was created, and it is presented in Figure 3f, in which CDs provide more nucleation sites to contribute to the interfacial interactions with PVDF molecular chains, facilitating the increased *β* crystalline phase and crystallinity degree, as well as a decreased crystallize size in CD/PVDF nanocomposites.

### 3.3. Frequency Dependence of Dielectric Property in a Low Electric Field

**Figure 4:**
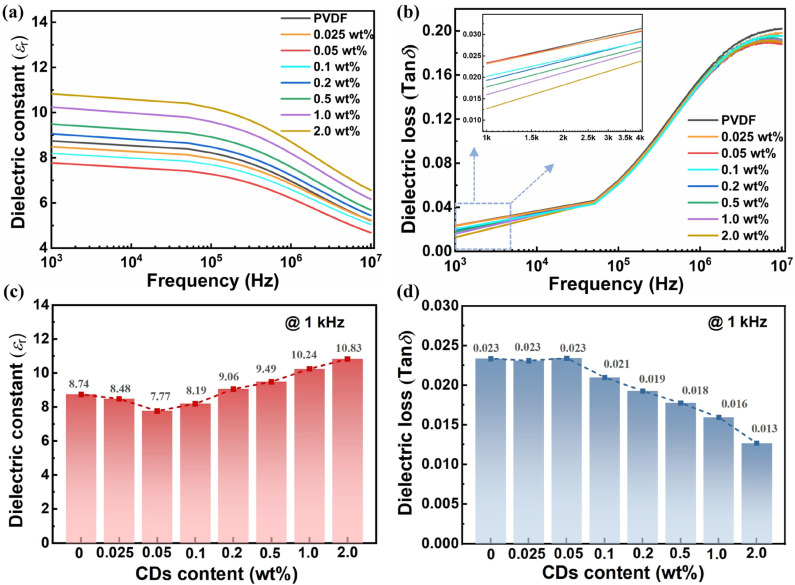
Frequency-dependent changes in (**a**) dielectric constant and (**b**) dielectric loss of CD/PVDF nanocomposites with different CD contents. Comparison of (**c**) dielectric constant and (**d**) dielectric loss at 1 kHz.

The dielectric properties as a function of frequency were further analyzed, and they are discussed in this section. As is known, the polarization behavior is closely related to the molecular chain movement and the frequency of the applied electric field for polymer dielectrics. Given resonance and relaxation regimes, dipole and interface polarization are two dominant polarization mechanisms for nonlinear PVDF polymer-based nanocomposite in the ranges from 1 kHz to 10 MHz [3]. With an increasing frequency, dipole orientation cannot catch up with the rapidly changing frequency of the applied electric field, which accounts for a downward trend in the dielectric constant and a simultaneous ascension in the dielectric loss, as illustrated in Figure 4a,b. Additionally, the dielectric constant and loss at 1 kHz were compared to clarify the effect of CDs’ incorporation on the dielectric property. In Figure 4c, as the CD content increases from 0 to 2 wt%, the dielectric constant shows a slight decrease, followed by a gradual increase. The initial slight decline at 0.05 wt% may be attributed to CDs hindering the chain segmental motion of the polymer matrix and suppressing dipole polarization, thus reducing the dielectric constant of the nanocomposite. When the CD content was continually increased compared to 2.0 wt%, the dielectric constant was elevated to 10.83, showing a 23.9% improvement to that of pristine PVDF (8.74). The promoting effect of CDs mainly manifests in the following aspects: First, CDs induced the formation of more polar *β*-phases in PVDF polymer, thereby enhancing dipole polarization. Second, more interface regions were constructed between the CD/PVDF heterogeneous medium to accumulate space charges, thus strengthening interface polarization. In Figure 4d, a suppressed dielectric loss was observed with the incorporation of CDs, e.g., that of 0.023 for PVDF and 0.013 for 2 wt% CD/PVDF nanocomposite, respectively, which is mainly attributed to the coulomb-blockade effect of CDs. The quantum-sized CDs could create an energy barrier-impeding electron motion through the polymer matrix, thereby reducing the conductive loss.

### 3.4. Weibull Analysis of Electric Breakdown Strength

**Figure 5:**
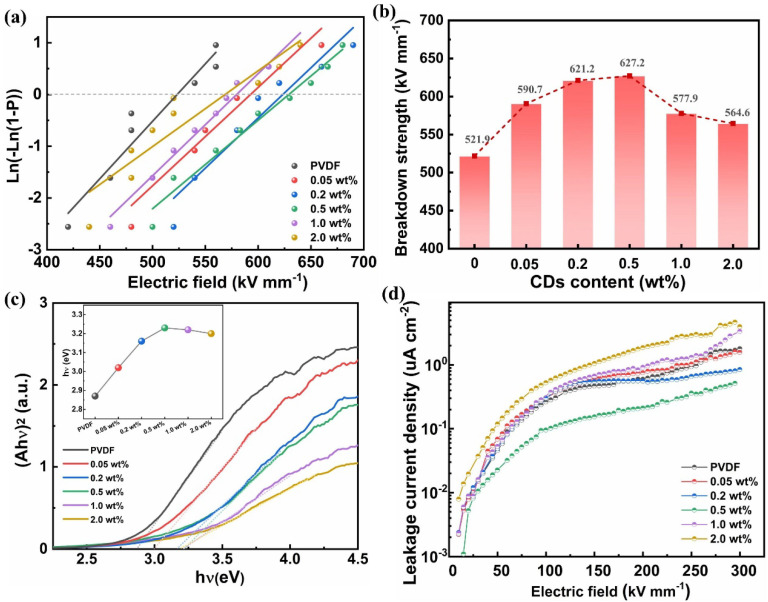
(**a**) Weibull distribution of the breakdown strength and (**b**) comparison of *E*_b_ of the CD/PVDF nanocomposites. (**c**) (*αhν*)^2^-h*ν* curves obtained from the UV−vis spectra and (**d**) the leakage current density of the CD/PVDF nanocomposites.

As shown in Figure 5a, the breakdown strength of the CD/PVDF nanocomposite was characterized by a two-parameter Weibull statistical analysis according to the following formula:

(7) $$P\left(E\right)=1-exp(-({E/E_{b})}^{\beta })$$

where *E*_b_ and *β* are the characteristic breakdown strengths for a specimen with a 63.2% breakdown probability and shape parameter reflecting the sample data dispersion, respectively. In Figure 5b, with increasing CD loading, the *E*_b_ of nanocomposites shows a trend of first increasing and then decreasing, and it reaches the maximum value at 0.5 wt% CDs. Notably, 0.5 wt% CD/PVDF nanocomposite exhibited a significantly improved *E*_b_ of 627.2 kV mm^−1^ compared with that of 521.9 kV mm^−1^ for PVDF. Here, multiple aspects were comprehensively considered to clarify the enhancement mechanism. As is well known, electromechanical failure and electrical conduction, respectively relating to mechanical strength and thermal breakdown, have been regarded as the two main breakdown mechanisms of dielectrics. For one thing, the *β*-PVDF possesses a much higher theoretical Young’s modulus of 287 GPa than that of 148 GPa for *α*-PVDF [12]. Also, compared with amorphous PVDF, the crystalline PVDF with a higher packing density and a lower free volume of polymer chains has a greater resistance to the spread of electronic trees. Therefore, the enhanced *β* phase and crystallinity induced via CDs endow the nanocomposites with excellent mechanical strength, showing a greater capability to hamper the onset of electromechanical failure. On the other side, the strong coulomb repulsion between electrons could form an energy barrier to generate the coulomb-block effect when the filler size is reduced to the quantum scale [32]. Thus, quantum-sized CDs could serve as deep trapping sites to depress the transport and movement of charge carriers. Furthermore, the energy band gap (*E*_g_) characterized by UV-Vis absorption spectra shows that CDs effectively modulate the energy level of the nanocomposite. Here, *E*_g_ is determined by the intersection of the tangent line and the horizontal axis. An increased *E*_g_ was observed from 2.87 eV for pristine PVDF to 3.23 eV for the 0.5 wt% CD/PVDF nanocomposite, which is beneficial for suppressing the charge injection from the electrode (Figure 5c). However, this enhancement effect on *E*_b_ has a critical threshold point for CD contents; e.g., when the CD content was above 0.5 wt%, the *E*_b_ declined instead. Excessive CDs would shorten the inter-filler distances and increase the probability of local filler aggregation, which makes it easier to form a conductive path and cause a leakage loss, thus resulting in a deteriorated *E*_b_. Finally, the high-field leakage behavior also confirms the above results. As shown in Figure 5d, at the same electric field of 300 kV mm^−1^, the leakage current density was reduced to 0.52 μA cm^−2^ for the 0.5 wt% CD/PVDF, compared with 1.80 μA cm^−2^ for pristine PVDF, and then followed by an increase to 4.67 μA cm^−2^ for the 2.0 wt% CD/PVDF nanocomposite.

### 3.5. High-Field Electric Polarization and Energy Storage Performance

**Figure 6:**
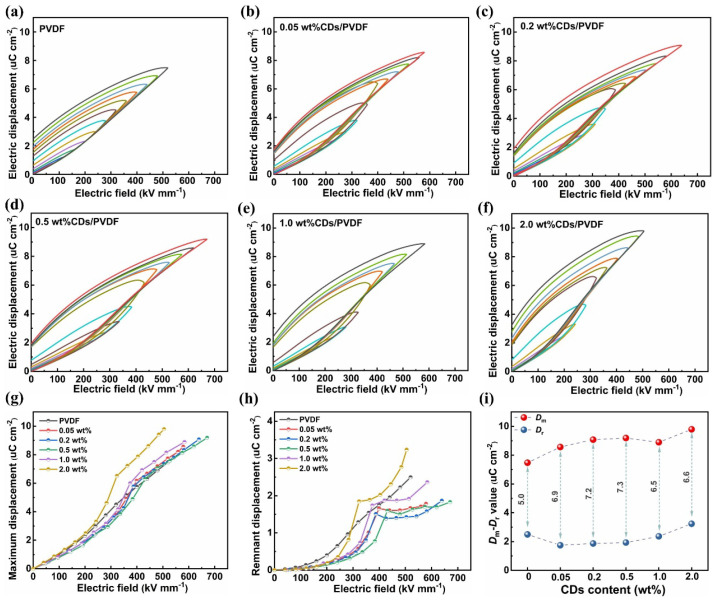
(**a**–**f**) High-field *D-E* loops of CD/PVDF nanocomposites with different CD contents. (**g**) *D*_m_ and (**h**) *D*_r_ of CD/PVDF nanocomposites under varied electric fields. (**i**) Displacement difference *D*_m_-*D*_r_ of CD/PVDF nanocomposites at maximum electric field.

The high-field polarization behavior of CD/PVDF nanocomposites was further analyzed using the typical electrical displacement-field hysteresis loop (*D-E* loop). The *D-E* loops of all samples under varied electric fields are presented in Figure 6a–f. The slenderer-shaped *D-E* loop represents the lower energy loss. Then, the maximum displacement, *D*_m_, and the remnant displacement, *D*_r_, of each sample film are summarized and compared in Figure 6g,h, respectively. As demonstrated, *D*_m_ gradually grows with an increasing electric field (Figure 6g) benefiting from the enhanced polarization. It is worth noting that, compared with the monotonically increased *D*_r_ for pristine PVDF, the variation in *D*_r_ in CD/PVDF nanocomposites is more complex. For instance, the *D*_r_ of CD/PVDF nanocomposites rises more rapidly at the electric field range from 200 to 400 kV mm^−1^, owing to an increased polar phase transition, and then remains at a stable plateau after polarization saturation. Subsequently, the higher field-induced conductivity loss raises the *D*_r_ again. Moreover, under the same electric field, the suppressed *D*_r_ is obtained in nanocomposites with the appropriate CD content. It is generally accepted that the polar *β* phase causes a high polarization loss and tends to increase the *D*_r_, which is not favorable to the energy discharge property. However, compared with pristine PVDF, the CD/PVDF nanocomposites with a larger *β*-phase exhibit a lower *D*_r_ instead, especially for the nanocomposites with CD content of 0.05–0.5 wt%. This is mainly attributed to the fact that the incorporation of CDs confines the charge carrier motion and reduces the leakage loss, leading to a decreased *D*_r_. When the CD content exceeds 1.0 wt%, the conductive path generated from the nanofiller aggregation of excessive CDs will cause a sharp increase in *D*_r_. Finally, the displacement difference *D*_m_-*D*_r_ at the maximum electric field for all samples is compared in Figure 6i. As the CD content increases, *D*_m_-*D*_r_ shows a trend of first rising and then descending. The highest *D*_m_-*D*_r_ value of 7.3 μC cm^−2^ was obtained with 0.5 wt% CD/PVDF nanocomposites, showing a 46% improvement compared to the pristine PVDF film (5.0 μC cm^−2^).

**Figure 7:**
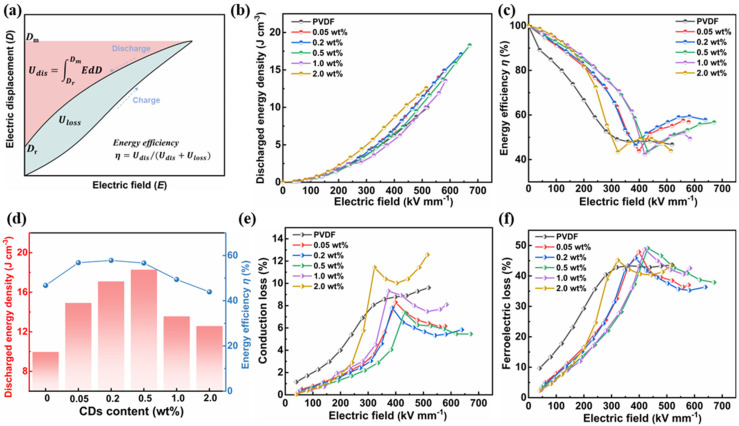
(**a**) Schematic *D-E* loop to calculate the energy storage performance. (**b**) The discharged energy density, *U*_dis_, and (**c**) energy efficiency, *η*, of the CD/PVDF nanocomposites under different electric fields. (**d**) Comparison of *U*_dis_ and *η* at the maximum electric field. (**e**) The conduction loss and (**f**) the ferroelectric loss of the CD/PVDF nanocomposites with varied electric fields.

The energy storage performance of nanocomposite was calculated by integrating the *D-E* loop using the following equation: $U_{dis}=\int_{D_{r}}^{D_{m}}EdD$, where *E* is the applied electric field, and *D*_m_ and *D*_r_ are the maximum and remnant electric displacement, respectively. Meanwhile, the area of the hysteresis curve represents the energy dissipation (*U*_loss_) caused by the polarization loss and conduction loss (Figure 7a). As presented in Figure 7b, the gradually continued enhancement in *U*_dis_ for all samples was obtained, benefiting from the intrinsic ability to allow greater polarization and storing more electrostatic energy with an increasing electric field. Compared with 9.9 J cm^−3^ at 519 kV mm^−1^ for pristine PVDF, the *U*_dis_ was elevated to 14.9 J cm^−3^ at 581.4 kV mm^−1^, 17.1 J cm^−3^ at 639.3 kV mm^−1^, 18.3 J cm^−3^ at 670.8 kV mm^−1^, 13.6 J cm^−3^ at 583.5 kV mm^−1^, and 12.6 J cm^−3^ at 514.8 kV mm^−1^ for the nanocomposites with CD content of 0.05, 0.2, 0.5, 1.0, and 2.0 wt.%, respectively. In particular, the highest *U*_dis_ of the 0.5 wt% CD/PVDF nanocomposites is 1.85 times higher than that of pristine PVDF. This significantly boosted energy density is mainly ascribed to the CD-induced synergistic effect of the increased polarization and higher breakdown. Additionally, the energy efficiency (*η*) evaluated using the formula $\eta =U_{dis}/\left(U_{dis}+U_{loss}\right)$ is shown in Figure 7c. Differing from the monotonic decrease in *η* in the pristine PVDF film, the CD/PVDF nanocomposite shows obvious superiority with the “*V*” shaped energy efficiency curves. The first decline in *η* at the relatively low electric field (less than 300–400 kV mm^−1^) is attributed to the polar phase transition, in which the irreversible dipoles cannot switch back when withdrawing the electric field, leading to the hysteresis *D-E* loop and increased *D*_r_. When the electric field was further raised, the unreversed dipoles increased slowly until reaching saturation. Comparatively, the newly reversible dipoles contributing to the discharging energy density increased quickly, inducing an ascension in *η.* For the nanocomposite with a high CD content of 1.0 and 2.0 wt%, the high field-induced leakage loss further led to another reduction in *η*. Finally, the *η* at the maximum electric field showed an obvious improvement from 46.8% for pristine PVDF to 57.8% for the 0.5 wt% CD/PVDF nanocomposite. Moreover, the energy loss mechanisms of the sample films were analyzed based on the *D-E* loops with the assumption that the measured displacement at the zero field (*D*_E=0_) mainly originates from the leakage current. The conduction loss, *η*_con_, was determined according to the following equation: $D_{E=0}=\eta_{con}E/2f$, where *f* is the frequency of the applied field [38]. Then, the ferroelectric loss, *η*_ferro_, could be obtained using the following formula: $\eta_{ferro}=1-\eta -\eta_{con}$. The conduction loss and ferroelectric loss with a varied electric field are shown in Figure 7e,f, respectively. Overall, the ferroelectric loss is much higher than the conduction loss at the same electric field, indicating its dominant role in energy loss mechanisms for a PVDF-based nanocomposite. Moreover, in Figure 7e, with an increasing CD content from 0 to 0.5 wt%, an obvious suppressed conduction loss is observed, e.g., from 9.6% for PVDF to 5.4% for the 0.5 wt% CD/PVDF nanocomposite at each *E*_b_, proving that the introduction of appropriate CDs effectively reduces the charge migration and leakage loss inside the nanocomposite film.

To sum up, the entire set of electrical performances was summarized and compared in Table 1 to assess the impact of CDs in nanocomposites. With a relatively moderate CD content (0.05–0.5 wt.%), *ɛ*_r_ and *D*_max_-*D*_r_ show a consistent upward trend. Also, *Tan* δ is suppressed while accompanied by the improved *E*_b_. Additionally, it should be noted that the maximum *ɛ*_r_ and minimum *Tan* δ are observed in the 2.0 wt% CD/PVDF nanocomposite, while the optimal *E*_b_ and *D*_max_-*D*_r_ are yielded in the 0.5 wt% CD/PVDF nanocomposite. This inconsistency in the CD content threshold is attributed to the different measurement conditions. It is known that the dielectric property is characterized under low-field testing (i.e., under an AC voltage of only 1.0 V_rms_). By comparison, *E*_b_ and *D*_max_-*D*_r_ are measured under high electric fields of several hundred kV/mm, which can more easily induce carrier migration and charge leakage. Therefore, a high field amplifies conductive loss, resulting in a lower CD content threshold for *E*_b_ and *D*_max_-*D*_r_ than for *ɛ*_r_ and *Tan* δ. Finally, energy density is dominated by a high-field *D-E* loop; thus, the 0.5 wt% CD/PVDF nanocomposite yields the optimal energy storage performance.

**Figure 8:**
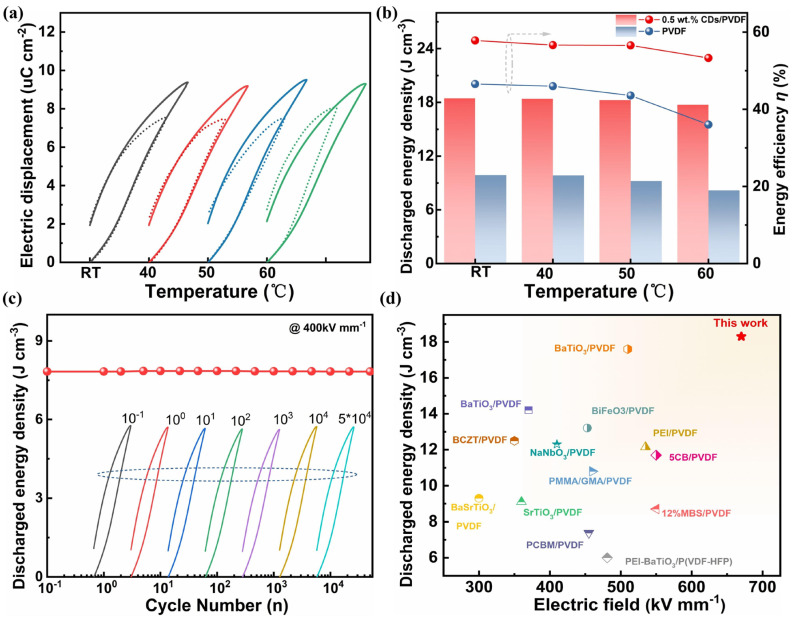
Temperature-dependent energy storage performance: (**a**) the *D-E* loop and (**b**) the discharged energy density and energy efficiency at different temperatures. (**c**) The charge–discharge cycle performance of pure PVDF and composites of the 0.5 wt% CD/PVDF nanocomposite. (**d**) A comparison of the *E*_b_ and the corresponding *U*_dis_ in this work with those of other recently reported PVDF-based nanocomposites [12,18,29,39,40,41,42,43,44,45,46,47,48,49,50].

Furthermore, as an important parameter for practical applications, temperature stability was assessed by testing the *D-E* loop of pristine PVDF (dashed line) and the 0.5 wt% CD/PVDF nanocomposite (straight line) at different temperatures, as shown in Figure 8a. With an increasing temperature from RT to 50 °C, *D-E* loops generally did not show a significant change difference, indicating excellent stability in this temperature range. When the temperature was further raised to 60 °C, a widening-shaped *D-E* loop was obtained in the pristine PVDF film. This is attributed to the thermal activation accelerating the carrier transport and increasing the leakage losses. By comparison, a relatively stable *D-E* loop was still maintained in the 0.5 wt% CD/PVDF nanocomposite film, indicating that the addition of carbon dots acting as charge traps restricts the excitation of charges and has a certain ability to resist thermal activation. Furthermore, the energy storage performance evaluated based on the *D-E* loop is shown in Figure 8b. With the increase in temperature from RT to 60 °C, the energy density and efficiency changed from 9.88 J cm^−3^ and 46.55% to 8.18 J cm^−3^ and 36.1% for pristine PVDF and from 18.46 J cm^−3^ and 57.8% to 17.74 J cm^−3^ and 53.3% for the 0.5 wt% CD/PVDF nanocomposite, respectively. The higher energy density and the higher efficiency retention ratio of 96.1% and 92.2% were present in the 0.5 wt% CD/PVDF nanocomposite, compared with 82.8% and 77.5% in the pristine PVDF film. In addition, the cycling stability of the dielectric film was evaluated at an electric field of 400 kV mm^−1^. As shown in Figure 8c, there is almost no observable attenuation in the U*_dis_* of the 0.5 wt% CD/PVDF nanocomposite after long-term 50k cycles, indicating the excellent stability and durability of this film. Finally, Figure 8d summarizes and compares the *E*_b_ and *U*_dis_ of the 0.5 wt% CD/PVDF nanocomposite films with those of recently reported PVDF-based dielectrics. The excellent performance produced in our work exceeds that of most analogous nanocomposites, indicating a favorable application for dielectric capacitors.

## 4. Conclusions

In this work, all-organic carbon quantum dots were incorporated into a PVDF polymer to significantly enhance the energy storage performance of the dielectric nanocomposite. A microstructural characterization proved that the ultrasmall and surface functionalized CDs act as nucleating inducers to facilitate the polar *β*-phase transition and crystallinity while decreasing the crystalline size of the PVDF polymer. Moreover, the appropriate introduction of CDs effectively modulates the energy level and traps of the nanocomposite, leading to an increased energy band gap, *E*_g_. An electrical property analysis revealed that the CD-induced polar transition and interface polarization contribute to improving *ε*_r_ and high-field *D*_m_. Additionally, a suppressed dielectric loss and high-field *D*_r_ were observed in the CD/PVDF nanocomposite due to the introduction of deep trap levels, effectively reducing conductive losses. More importantly, *E*_b_ was largely increased from 521.9 kV mm^−1^ for PVDF to 627.2 kV mm^−1^ for the 0.5 wt% CD/PVDF nanocomposite, which was attributed to the construction of a higher energy barrier and deeper traps inside the nanocomposite to capture charges and reduce the transmission of charge carriers. As a result, benefiting from improved *E*_b_ and *D*_m_, as well as the simultaneous reduction in *D*_r_, the 0.5 wt% CD/PVDF nanocomposite achieved the highest discharge energy density of 18.3 J cm^−3^ with an efficiency of 56.7%, showing a significant improvement compared with that of 9.9 J cm^−3^ and 46.8% for pristine PVDF. The excellent energy storage performance is comparable with most analogous PVDF-based nanocomposites. This study demonstrates that a new strategy of organic quantum dot-enhanced energy storage performance in PVDF-based dielectric capacitors will promote its application for advanced electrical and electronic devices.

## Figures and Tables

**Table 1:** Comparison and summary of the entire set of electrical performances.

Dielectrics	*ɛ*_r_	*Tan* δ	*E*_b_ (kV mm^−1^)	*D*_max_-*D*_r_ (μC cm^−2^)	*U*_e_ (J/cm^−3^)	*η*
0	8.74	0.023	521.9	5.0	9.96	46.76
0.025 wt%	8.48	0.023	-	-	-	-
0.05 wt	7.77	0.023	590.7	6.9	14.91	56.81
0.1 wt%	8.19	0.021	-	-	-	-
0.2 wt%	9.06	0.019	621.2	7.2	17.09	57.82
0.5 wt%	9.49	0.018	627.2	7.3	18.28	56.67
1.0 wt%	10.24	0.016	577.9	6.5	13.56	49.37
2.0 wt%	10.83	0.013	564.6	6.6	12.57	43.91

## Data Availability

Data are contained within the article.
